# Fluid confinement within a branched polymer structure enhances tribological performance of a poly(2-methacryloyloxyethyl phosphorylcholine)-surface-modified contact lens

**DOI:** 10.1098/rsos.240957

**Published:** 2024-10-02

**Authors:** Vinay Sharma, Xinfeng Charlie Shi, George Yao, Ying Zheng, Nicholas D. Spencer, James Yuliang Wu

**Affiliations:** ^1^ Alcon Research, LLC, Fort Worth, TX 76134, USA; ^2^ Alcon Research, LLC, Duluth, GA 30097, USA; ^3^ Department of Materials, ETH Zürich, 8093 Zürich, Switzerland

**Keywords:** fluid, confinement, branched, polymers, structures, enhances

## Abstract

The poly(2-methacryloyloxyethyl phosphorylcholine) (PMPC)-modified, silicone hydrogel, contact lens (CL) material *lehfilcon A* has previously been demonstrated to have a lubricious, antifouling and ultra-soft surface. This study provides confirmatory identification of the outer polymer structures on this CL surface as branched PMPC structures. It further aims to understand their role in providing enhanced tribological performance via fluid confinement. A combination of scanning transmission electron microscopy and atomic force microscopy infrared spectroscopy has been used to achieve both morphological and chemical confirmation of branched PMPC structures resembling the polysaccharide species present on the surface of the cornea. Measurements of the fluid-confinement behaviour of this layer, by means of nanoindentation experiments, show it to resist squeeze-out of the interstitial fluid, thereby boosting lubrication by virtue of a fluid-load-support mechanism. Tribological testing of CLs showed this effective lubrication to be maintained after one month of daily wearing.

## 1. Introduction

**Figure 1.**
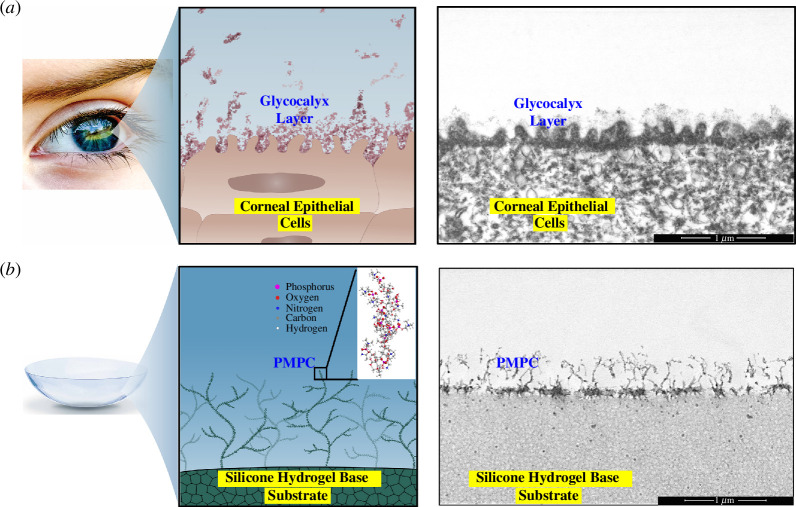
Biomimetic engineered surface is inspired by the corneal epithelial surface. (*a*) Schematic diagram and STEM image showing the glycocalyx layer on the surface of human corneal epithelial cells. (*b*) Schematic diagram and STEM image of a PMPC-surface-modified *lehfilcon A* contact lens.

The potential applications of poly(2-methacryloyloxyethyl phosphorylcholine) (PMPC) in the field of medical devices are well documented in the literature [1–4] owing to its bioinspired and biocompatible nature. In our previous work, we showed that interfacial compatibility between natural tissues and artificial medical devices can be achieved through biomimetic material design [5]. We highlighted the modification of the silicone hydrogel (SiHy) contact lens (CL) by means of PMPC, which creates a surface similar to that of the human cornea in terms of both morphology and mechanical properties [6]. Such a unique biomimetic contact lens material, *lehfilcon A*, presents itself as a prime example of a medical device into which PMPC chemistry has been successfully integrated on an industrial scale. In addition, it has demonstrated long-lasting, superior performance in both laboratory and clinical settings [7–9]. Figure 1 presents a schematic with scanning transmission electron microscopy (STEM) images of a cornea and a *lehfilcon A* CL, showing the remarkable resemblance between features evident on the two surfaces.

Lee & Spencer [10] have argued that biomimetic design is the key to attaining practical implementation of water-based lubrication for synthetic polymers. Inspired by the lubrication mechanisms observed in nature, many tribological studies have been conducted for soft materials grafted with polymer brushes or large mesh size hydrogels, that significantly reduce interfacial friction during sliding [11–18]. This effect depends on a combination of two important surface properties of the materials involved. The first is the low modulus of at least one of the surfaces, so that it can deform elastically to reduce the contact pressure against an acting force [19]. The second is the fluid retention or holding capability of the ‘hairy’ polymers at the surface [20], which assists lubrication by a mechanism in which the confined motion of the fluid through the brush leads to partial support of the applied load (fluid-load support) [21]. In our previous works, we have highlighted the biomimetic design and surface properties of the *lehfilcon A* CL, which includes the bioinspired PMPC chemistry, with its antifouling capability, as well as its visibly branched and potentially entangled, corneal-tissue-mimicking surface morphology and elastic modulus [5,6,22].

In this study, we provide additional evidence of the chemical nature, location and thickness of the branched PMPC layer by means of atomic force microscopy-infrared spectroscopy (AFM-IR) measurements, which allow direct imaging of the polymer structures on the surface. Moreover, AFM nanoindentation experiments have enabled us to demonstrate the fluid-confining capability of the PMPC-modified surface under load at high indentation rates. The branched PMPC-grafted SiHy that comprises the *lehfilcon A* CL appears to achieve its superior tribological performance by combining both characteristics—low modulus and fluid retention—required to exhibit effective, water-based lubrication. Furthermore, this lubricity appears to be maintained after one month of daily wearing.

## 2. Experimental

### 2.1. Materials

The two samples used in this study comprised a PMPC-surface-modified *lehfilcon A* CL (Alcon, Fort Worth, TX, USA) and its SiHy base substrate. Details regarding the monomers and their processing have been extensively discussed in our previously published work [5,6,22]. Briefly, for surface-modified *lehfilcon A*, an initial interpenetrating anchoring layer was prepared on the surface of a cured base substrate by dipping it into a solution of poly(methacrylic acid). Then, the base substrate was immersed into a premixed aqueous solution of poly(amidoamine-epichlorohydrin) and reactive MPC (2-methacryloyloxyethyl phosphorylcholine) polymers to produce a PMPC layer at the surface.

### 2.2. Clinical samples

Five PMPC-surface-modified *lehfilcon A* CLs from an Alcon clinical study were obtained after one month of daily wearing and cleaning for tribological testing. The clinical study was carried out in compliance with the Declaration of Helsinki. Informed consent was given and signed by all participants prior to enrollment in the study.

### 2.3. Tribological testing

The tribological testing of the CL samples in this study was conducted with a NTR2 Nano Tribometer (Anton Paar, Graz, Austria), which has a high-resolution, piezo-controlled, linear-reciprocating stage. The counter surface for these experiments was prepared by attaching a probe made of super-cushioning, abrasion-resistant polyurethane rubber sheet (1.0 mm thick; durometer 4000; McMaster-Carr, CA, USA) to the force sensor. A custom-designed, dome-shaped lens holder was used here for tribological testing. A doughnut-shaped top fixture of the holder clamped the sample in place using small magnets. The hole in the centre gave access to the sample for testing and provided a fluid reservoir to maintain full hydration of the CL sample, which remained in contact with its packaging solution as it reciprocated against the stationary, soft-rubber counter-surface. The stage was reciprocated at 0.1 Hz and the stroke length was kept at 0.5 mm. The normal load was set to change incrementally from 0.25 to 1 mN in five steps. A total of 30 cycles for each sample were run with six cycles at each incremental load. For data processing, the middle 0.1 mm portion of the forward and reverse strokes of the tangential force hysteresis loop were used and the mean coefficient of friction (CoF) values were calculated by the instrument software. The CoF data resulting from five samples of each lens type were used to calculate the average and standard deviation. In addition, one-way ANOVA statistical analysis (for *p*‐values) was conducted for the CoF results using Minitab software.

### 2.4. Scanning transmission electron microscopy imaging

The *lehfilcon A* CL and human cornea samples were imaged in cross-section using an FEI Quanta 250 field-emission scanning electron microscope system with a STEM detector at an accelerating voltage of 30 kV in low-vacuum mode. The 70 nm thick cross-sections of these samples were cut using a PowerTome PCZ ultramicrotome (Boeckeler Instruments, Inc., Tucson, AZ, USA). The sample-preparation process, which involves RuO_4_ staining for differential contrast and structure preservation, alcohol dehydration and resin embedding, has been explained in detail in our previous work [6].

### 2.5. Atomic force microscopy-infrared spectroscopy imaging

AFM-IR imaging allows different materials with distinct IR absorption peak positions to be chemically mapped within the same sample. First, a tunable laser source is set to an IR wavenumber that is known to be absorbed specifically by the material of interest. Then, the sample is scanned by the AFM probe with the IR laser focused immediately underneath the probe tip. When the probe is placed directly on top of the material of interest, the thermal expansion induced by absorption of the IR laser radiation results in cantilever deflection. Thus, by scanning the sample under the AFM probe and laser, the cantilever deflection can be used to generate an IR-absorption map of the scanned area at the selected wavenumber [23].

A NanoIR3 (Bruker Nano, Santa Barbara, CA, USA) system equipped with a quantum cascade laser (QCL) source (780–1800 cm^−1^) was used in contact AFM-IR mode to conduct nanoscale chemical imaging. The experiments were conducted using Bruker’s PR-EX-NIR2-10 contact-mode probe (resonant frequency 13 ± 4 kHz) and a 70 nm thick cross-section of the CL sample mounted on a gold-coated silicon wafer. This thin cross-section was prepared using the same sample and procedure described above in §2.4. A thin section from ultramicrotome’s diamond knife boat was lifted using a metallic Perfect Loop (Electron Microscopy Sciences, PA, USA). The loop was slowly lowered over the thin section and put in contact with the water without breaking the surface tension of the water. Once the section was held within the loop by a thin film of water it was transferred onto the gold-coated silicon wafer. For AFM-IR imaging, the peak positions for PMPC and polydimethylsiloxane (PDMS) were selected based on the literature [24–27]. Both 800 cm^−1^ (Si–C stretching in PDMS) and 967 cm^−1^ (P–O stretching in PMPC) wavenumbers were used to generate 5 µm × 5 µm IR chemical images at a scan rate of 0.5 Hz for PDMS and PMPC, respectively.

### 2.6. Atomic force microscopy nanoindentation

**Figure 2.**
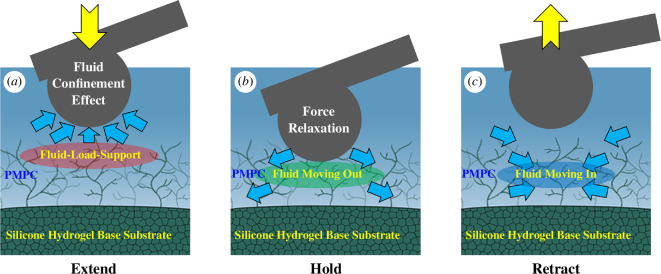
AFM nanoindentation ramp-script steps: (*a*) extend Z-position of the piezo to reach the force setpoint of 6 nN; (*b*) hold Z-position of the piezo for 1 s for force relaxation; and (*c*) retract Z to home position.

The AFM nanoindentation experiments for the *lehfilcon A* CL and SiHy base substrate samples were conducted using a Dimension FastScan Bio Icon Atomic Force Microscope (Bruker Nano, Santa Barbara, CA, USA) operating in the ‘PeakForce QNM in Fluid’ mode. It has previously been shown that fluid confinement by polymer brushes can be studied by conducting nanoindentation with a relatively large spherical probe at varying indentation rates [21,28]. We used the same approach in this work for nanoindentation of a PMPC-surface-modified *lehfilcon A* CL and the monitoring of fluid confinement. A SAA-HPI (Bruker) 7 µm diameter probe with a unique hemispherical tip and a spring constant of 0.22 N m^−1^ was used to conduct the study. During experiments, the samples were mounted on the same custom-designed, dome-shaped lens holder as described earlier. At a fixed force set-point of 6 nN, a series of indentations was carried out at different indentation rates for both a *lehfilcon A* CL (0.2, 4, 8, 20 and 40 µm s^−1^) and a SiHy base substrate (0.2 and 4 µm s^−1^) sample in lens-packaging solution. An SAA-HPI (Bruker) 7 µm diameter probe was used for these experiments as well. The CL was also subjected to force-relaxation experiments in fluid using the ‘Ramp Script’ function in the AFM software, which allowed the Z position of the piezo to be maintained for 1 s after reaching the desired force set-point. The script included three steps: (i) extend Z-position of the piezo to reach the force set-point of 6 nN; (ii) hold Z-position of the piezo for 1 s; and (iii) retract Z to home position as shown in figure 2. In a series of experiments, the tip velocity in step (i) of the script was set to 0.2, 4, 8 and 10 µm s^−1^ to capture force-relaxation data at different indentation rates. The maximum tip velocity was limited to 10 µm s^−1^ by the ramp-script function.

## 3. Results and discussion

### 3.1. Tribological performance

**Figure 3.**
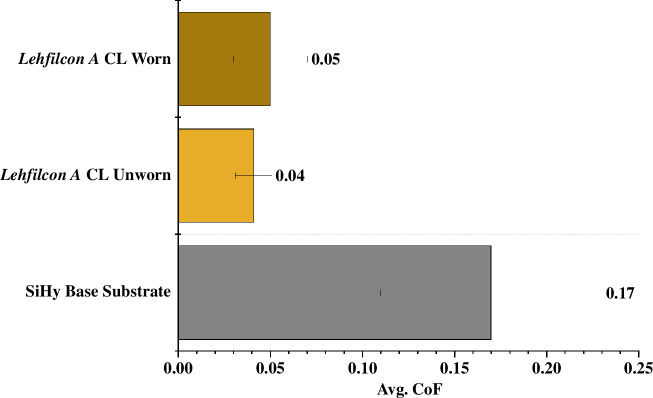
Graph showing average coefficient of friction (Avg. CoF) data for worn and unworn *lehfilcon A* CL and, SiHy base substrate samples when slid against a very soft polyurethane rubber surface under an incremental load of 0.25–1 mN in lens-packaging solution.

Figure 3 shows the results of the experimental evaluation of the tribological performance of a worn *lehfilcon A* CL, unworn *lehfilcon A* CL and SiHy base substrate (*n* = 5 for all) when evaluated against a very soft polyurethane rubber surface in lens-packaging solution. The experimental set-up was designed to exert the normal load with the stationary rubber probe, while the contact lens sample was reciprocated against it along a linear stroke length. The testing parameters were chosen to achieve contact pressure within the 2–10 kPa range which is comparable to eyelid pressure during blink. The average CoF (±s.d.) for worn *lehfilcon A* CL, unworn *lehfilcon A* CL and SiHy base substrate were measured to be 0.05 (±0.02), 0.04 (±0.01) and 0.17 (±0.06), respectively. One-way ANOVA statistical analysis showed that the average CoF values of worn and unworn *lehfilcon A* CL samples were significantly (*p < *0.05) lower than that of SiHy base substrate. In addition, the worn and unworn *lehfilcon A* CL samples did not have any significant (*p > *0.05) difference in their average CoF values.

### 3.2. Atomic force microscopy-infrared spectroscopy chemical imaging

**Figure 4.**
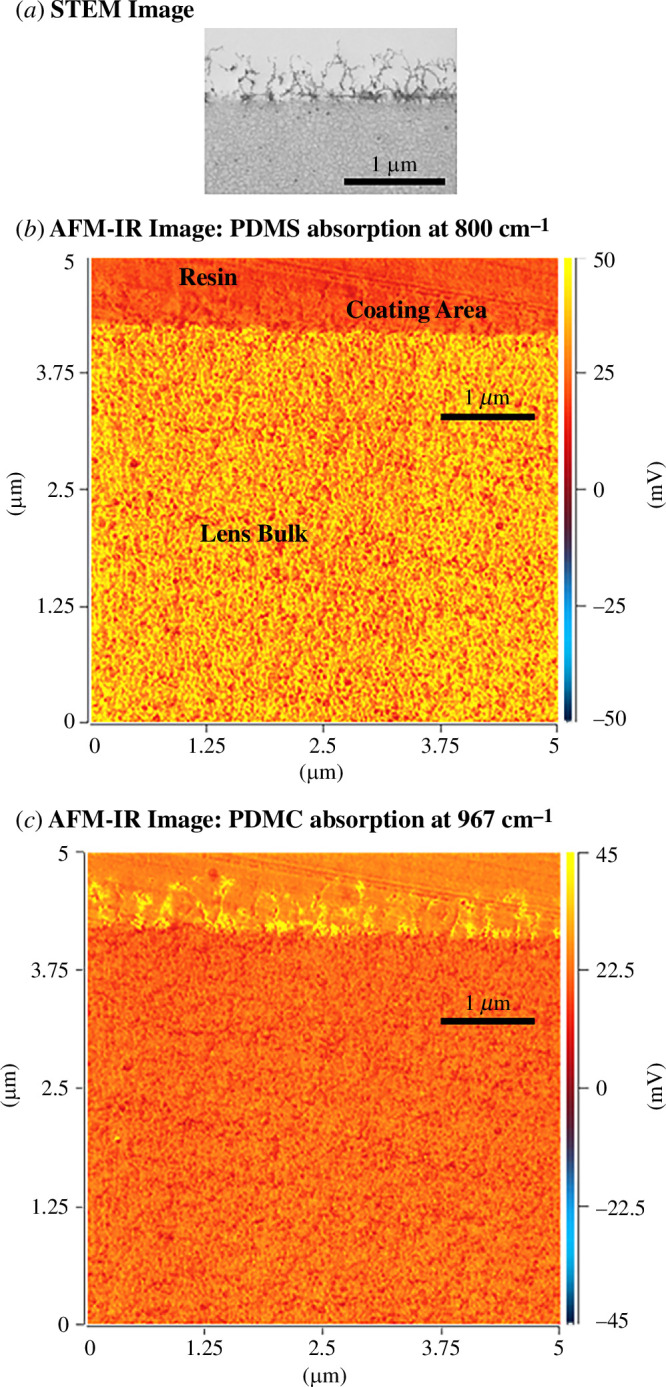
STEM and AFM-IR cross-sectional images for a *lehfilcon A* CL following RuO_4_ staining, alcohol dehydration and resin embedding. (*a*) STEM image showing branched polymer structures at CL surface; (*b*) AFM-IR image at 800 cm^−1^ showing PDMS presence in the lens bulk; (*c*) AFM-IR image at 967 cm^−1^ showing PMPC presence at the CL surface. Yellow colour denotes the presence of the specific IR signal.

Figure 4*a* shows the STEM image from a 70 nm thick cross-section of a *lehfilcon A* CL sample for comparison, as the AFM-IR imaging was conducted on a similar thin cross-section from the same sample. Both the AFM-IR images (figure 4*b*,*c*) are displayed in the ‘Hot-Cold’ colour scheme and the colour-scale bars are based on the IR amplitude and centered at zero. Yellow in the IR image corresponds to the presence of specific chemical bonds absorbing at the selected wavenumber, while shades of orange represent areas of their absence. AFM-IR imaging (at 967 cm^−1^ for P–O stretching) of a cross-section of a biomimetic *lehfilcon A* CL (figure 4*c*), therefore, clearly shows the presence of PMPC chemistry and the branched and potentially crosslinked or entangled structure at the CL surface, matching the shape, form and thickness of the structures visible in the STEM image of the same sample. In addition, the PDMS in the bulk of the SiHy base substrate was also characterized by imaging the 800 cm^−1^ peak for Si–C stretching (figure 4*b*). No signal for PDMS was observed at the surface of the CL interfacing with the resin. Thus, the AFM-IR image (figure 4*c*) allows us to chemically map the branched polymer structures on the surface of the *lehfilcon A* CL and confirm that they are indeed characteristic of PMPC chemistry. As expected, the PMPC signal is only observed at the interface between the lens’s bulk and the resin.

### 3.3. Atomic force microscopy nanoindentation and fluid confinement by poly(2-methacryloyloxyethyl phosphorylcholine) layer

**Figure 5.**
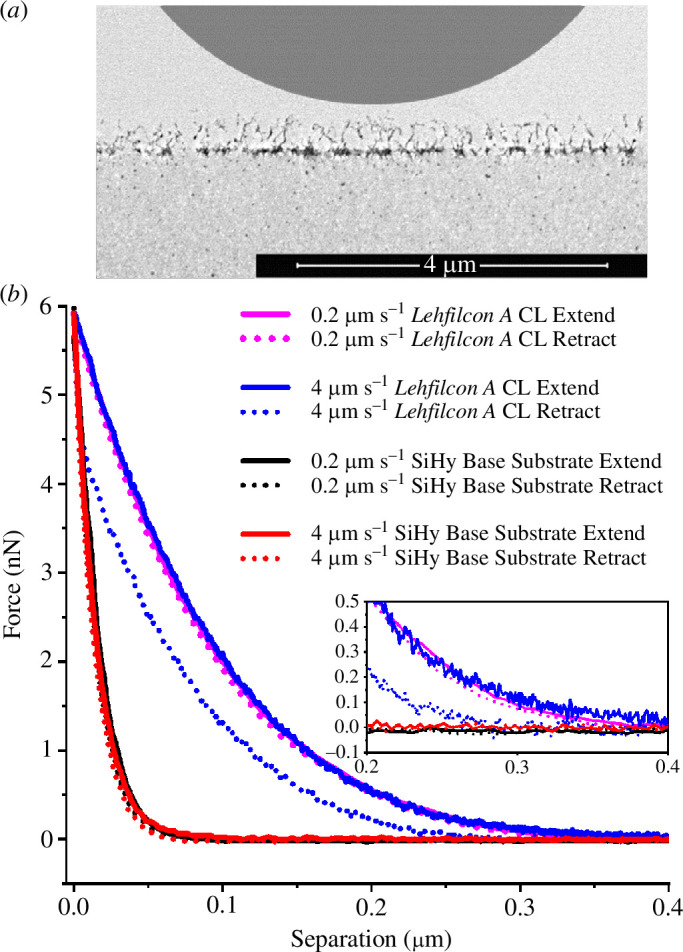
(*a*) Schematic indicating the size of 7 µm diameter AFM probe tip relative to the PMPC structures at the surface of a *lehfilcon A* CL; (*b*) Indentation force curves, highlighting the differences in the mechanical properties of a *lehfilcon A* CL and a SiHy base substrate.

Figure 5*a* is a schematic, drawn to scale, to show the size of the 7 µm diameter AFM probe tip relative to the surface structures visible in the STEM image of a PMPC-surface-modified *lehfilcon A* CL. It indicates that the size of the tip is significantly larger than the branched PMPC surface structures. This relative size difference between the surface structures and the probe diameter is critical in the observation of the fluid-confinement behaviour. Figure 5*b* shows indentation force-curves for a *lehfilcon A* CL and a SiHy base substrate at 0.2 and 4 µm s^−1^ indentation rates. The extended curves in the plot are shown with ‘solid’ lines, while the ‘short-dashed’ lines denote the retract curves. The difference between the two materials (PMPC layer on CL or SiHy base only) can be related to their different porous structures and chemistries, which can influence hydrophilicity. This is reflected in their mechanical properties, which are evident in the shapes of their respective force curves. Owing to the presence of the PMPC layer at the surface of the *lehfilcon A* CL, the force curves have an early onset of contact and show an indentation depth that is consistent with the PMPC-layer thickness observed in both STEM and AFM-IR (figure 4) and significantly deeper than that observed for the SiHy base substrate at the same applied load.

In our previous work, we conducted extensive surface-modulus characterization of these two materials and showed how the modulus changes as a function of indentation depth [29]. Figure 5*b* shows another important difference between the force curves for the two materials that is primarily observed at faster indentation rates for a *lehfilcon A* CL. The extend and retract curves for a *lehfilcon* A CL at 0.2 µm s^−1^ overlap each other completely but at 4 µm s^−1^ indentation rate they start to show separation. Here, figure 5*a* helps in understanding the interaction of the 7 µm diameter AFM probe tip with the branched PMPC polymer structures. At the slower 0.2 µm s^−1^ indentation rate, the fluid trapped between the PMPC structures has enough time to move out of the contact area and it does not exert any force in the opposite direction, whereas at the higher, 4 µm s^−1^ indentation rate we observe the fluid confinement, as previously described by Mathis *et al*. in their study of rate-dependent fluid confinement within thin surface-grafted polymer layers [23]. These nanoindentation results highlight the role of PMPC chemistry and structure in significantly altering the water-confining properties of the system following the surface modification of the SiHy base substrate.

**Figure 6.**
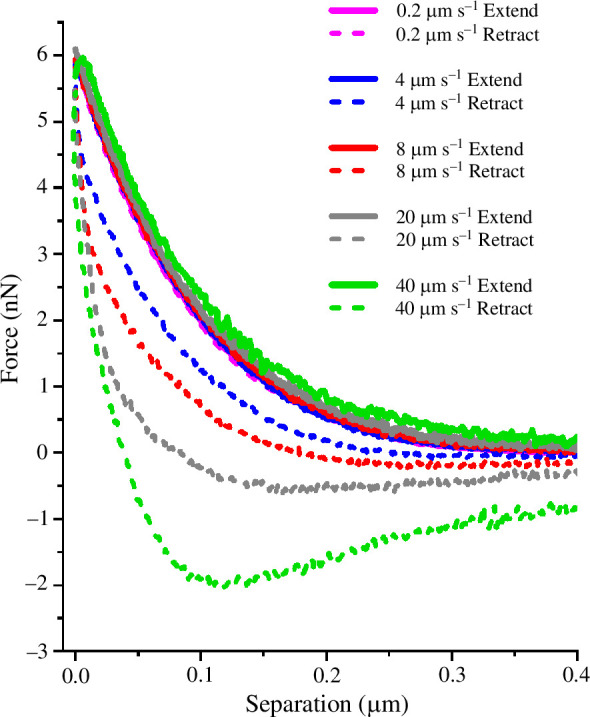
Indentation force curves demonstrating the amplified fluid confinement behaviour for a *lehfilcon* A CL surface at increasing indentation rates.

Experiments were carried out at very high indentation rates to more effectively reveal the fluid-confinement behaviour of the branched-polymer surface structures derived from PMPC. Figure 6 shows nanoindentation force curves recorded for a *lehfilcon A* CL at indentation rates ranging from lowest (0.2 µm s^−1^) to highest (40 µm s^−1^). The extend and retract curves at 0.2 µm s^−1^ show the baseline behaviour of the PMPC-modified surface and it is evident that the separation between extend and retract curves is increasing upon increasing the indentation rate (8, 20 and 40 µm s^−1^). The nature of these force curves with increasing force offset at zero indentation closely resembles behaviour reported by Mathis *et al*. for poly(dodecylmethacrylate) (P12MA) polymer brushes in hexadecane [21].

**Figure 7.**
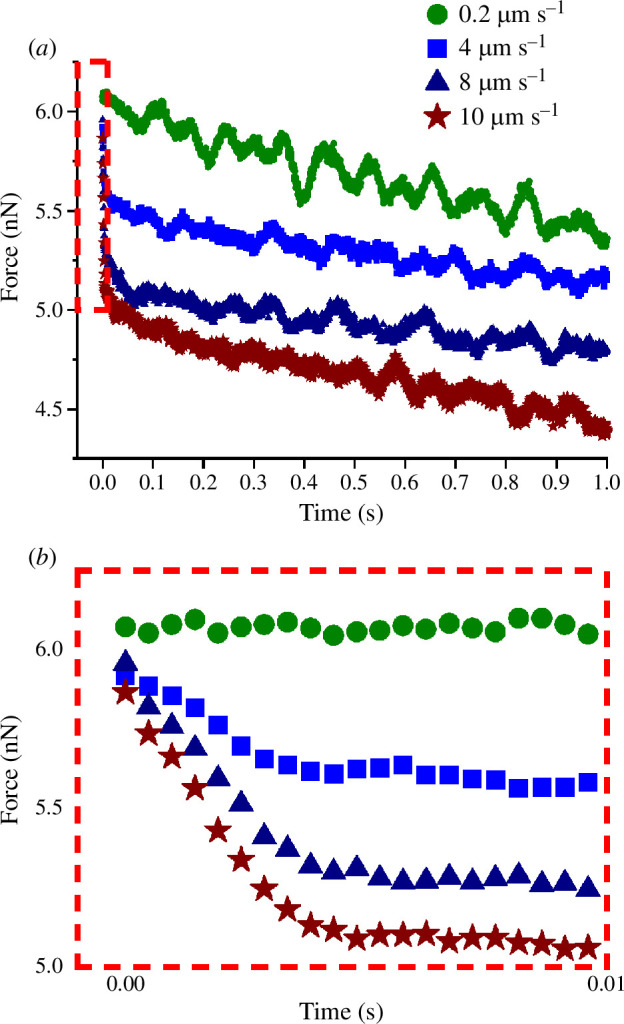
(*a*) Force-relaxation data for a *lehfilcon A* CL plotted as a function of time after reaching the load set-point at increasing indentation rates. (*b*) Zoomed-in view of the force-relaxation data showing the significant reduction of force at higher rates within first 0.01 s.

To further understand and confirm the confinement of fluid by branched PMPC polymer structures, a force-relaxation study at increasing indentation rates was also conducted for a *lehfilcon A* CL. These experiments involved holding the Z position of the piezo for 1 s after reaching the desired force set-point of 6 nN. Using this approach, indentations at 0.2, 4, 8 and 10 µm s^−1^ were carried out and the force-relaxation data for each indentation rate was plotted as a function of hold time, as shown in figure 7*a*. These data clearly show that the drop in force or the force-relaxation is least at the lowest rate and maximum at the highest rate. To further highlight the amplitude of this force-relaxation at the increasing rates, force values for the initial 0.01 s (i.e. within the red box in figure 7*a*) were replotted and are shown in figure 7*b* . These data reflect similar behaviour to that described by Ateshian [30] for cartilage and Mathis *et al.* [23] for polymer brushes, where they discuss the relationship between the advective and diffusive transport rates (known as the Péclet number) in confined-fluid systems. In cases where the diffusion of fluid away from the contact region cannot keep up with the arrival of fluid during loading, the fluid itself carries part of the load (fluid-load support), assisting the lubrication. In the data in figure 7, it appears that upon reaching the force set-point at 0.2 µm s^−1^ rate the fluid has had sufficient time to diffuse away from the contact area and hence there is no significant drop in the force after stopping the indentation. However, at higher rates, the fluid confined by the PMPC layer shares the load during indentation, and then continues to flow out of the contact area when the indentation is stopped. This leads to a substantial force-relaxation (approx. 1 nN in less than 0.01 s for 10 µm s^−1^) after stopping, as depicted in figure 7*b*. The force-relaxation data also demonstrate that the surface of the PMPC-modified *lehfilcon A* CL is capable of holding or confining significant fluid, which can share the applied load from a rapidly approaching agent of force or pressure. This mechanism may boost lubrication in, for example, a CL exposed to the force of an approaching eyelid during blinking. While this has not been previously reported for a branched polymer layer, there have been suggestions in the literature that crosslinked PMPC structures can display lower friction than their linear counterparts at low speeds [31].

Data supporting the results figures 1, 3–5 and figures 3–5 have been uploaded as the electronic supplementary material.

## 4. Conclusions

AFM-infrared measurements confirm STEM measurements in this and a previous study that the PMPC layer is present on the SiHy surface as a branched and/or possibly entangled layer. AFM indentation experiments highlight the considerably lower modulus of the PMPC layer in comparison to the SiHy substrate, and also show that the layer is capable of fluid-load support when subjected to a sufficiently rapid onset of load. Such a phenomenon is characteristic of systems such as cartilage or polymer brushes and is partially responsible for their low-friction behaviour.

Thus, it has been confirmed that the low-friction properties of the *lehfilcon A* CL can be attributed to the outer, branched and possibly entangled PMPC layer, whose hydrophilic nature and fluid-confining structure provide a degree of fluid-load support when subjected to external forces, eliciting a highly lubricious response to tribological challenges such as those imposed by an approaching eyelid.

## Data Availability

Data supporting the results figures 1, 3–5 and figures 3–5 have been uploaded as the electronic supplementary material [32].
